# Dynamic alterations in m^6^A RNA methylation profiles during involution of infantile hemangiomas

**DOI:** 10.3389/fonc.2025.1652621

**Published:** 2025-09-04

**Authors:** Pinru Wu, Qingqing Cen, Ying Shang, Junyan Liang, Gang Ma

**Affiliations:** ^1^ Department of Laser and Aesthetic Medicine, Shanghai Ninth People’s Hospital, Shanghai Jiao Tong University School of Medicine, Shanghai, China; ^2^ Department of Dermatology, Shanghai Ninth People’s Hospital, Shanghai Jiao Tong University School of Medicine, Shanghai, China; ^3^ Department of Plastic and Reconstructive Surgery, Shanghai Ninth People’s Hospital, Shanghai Jiao Tong University School of Medicine, Shanghai, China

**Keywords:** infantile hemangioma, m6A methylation, proliferative phase, involuting phase, RNA modification

## Abstract

**Introduction:**

Infantile hemangioma (IH) is a common benign vascular tumor characterized by a proliferative phase followed by regression. N6-methyladenosine (m6A) methylation, a major RNA modification, plays a critical role in tumor development, though its function in IH remains unclear.

**Methods:**

This study analyzed six IH samples (three from proliferative IH, three from involuting IH), using transcript-specific microarrays after m6A immunoprecipitation to explore dynamic methylation changes and their regulatory impact on gene expression.

**Results:**

Results showed significantly lower m6A levels in involuting-phase hemangiomas. Differentially methylated genes (DMGs) were mainly involved in biological processes such as cell-cell junction and cell-matrix adhesion. KEGG pathway analysis revealed DMGs were enriched in MAPK, Calcium, and PI3K-Akt signaling pathways, suggesting that m6A modifications are closely linked to angiogenesis and tumor growth. MeRIP-qPCR showed that IGF1 and IGF2 exhibiting significant correlation in both m6A levels and expression. The overall downregulation of m6A modification for lncRNA and sncRNA suggested active demethylation processes may involve in involution of IH.

**Discussion:**

Overall, this study demonstrates that m6A methylation modulates key cellular pathways in IH progression and may serve as a promising target for future diagnostic and therapeutic strategies.

## 1 Introduction

Infantile hemangioma (IH) is the most common benign vascular tumor, primarily occurring during the neonatal period (1). It follows a characteristic biphasic progression, within a few months after birth, it enters the proliferative phase, marked by rapid endothelial cell proliferation and angiogenesis (2). Subsequently, it transitions into the involuting phase, during which the tumor gradually shrinks and may partially or completely regress (3, 4). Although the natural progression of this condition is well-documented, the molecular mechanisms underlying its development and involution remain incompletely understood, particularly at the level of epigenetic regulation (5, 6). Current research indicates distinct gene expression profiles and epigenetic modifications between the proliferative and involuting IH, with differentially expressed genes (DEGs) critically regulating angiogenesis, cell proliferation, and apoptosis (7). A deeper understanding of these molecular alterations could elucidate the mechanisms driving hemangioma pathogenesis while potentially identifying novel diagnostic markers and therapeutic targets for clinical application.

N6-methyladenosine (m6A) modification represents one of the most prevalent RNA modifications, occurring extensively in both mRNA and diverse non-coding RNAs (8). This modification plays crucial regulatory role in multiple RNA processed, including stability, splicing, transport, and translation efficiency (9–11). Recent studies have demonstrated that m6A modification participates in diverse biological process, including gene expression regulation, embryonic development, cell fate determination, and immune responses (12). Furthermore, accumulating evidence reveals its significant association with the pathogenesis and progression of multiple diseases (13, 14). In cancer biology, m6A modification critically regulates tumor cell proliferation, migration, differentiation, and apoptosis, thereby playing a pivotal role in tumorigenesis, progression, and metastasis dissemination (15). Kun et al. found that HECW2 regulates the ubiquitination of ALKBH5, which subsequently enhances LDHA expression through m6A-mediated demethylation of LDHA mRNA, promoting the development of infantile hemangioma (16). Therefore, investigating the role of m6A methylation in the development of IH, especially its dynamic regulation during the proliferative-to-involuting phases transition, holds significant potential for elucidating the IH’s molecular mechanisms.

Previous studies have demonstrated that genes such as *HIF1A*, *IGF1*, and *IGF2* were upregulated during the proliferative phase of infantile hemangioma (17–21). HIF-1α was significantly overexpressed in IH tissues and hemangioma-derived endothelial cells at both mRNA and protein levels (21). Notably, propranolol treatment reduces HIF-1α expression in IH patients, and its overexpression reverses propranolol’s inhibitory effects on VEGF expression and cell proliferation (17). IGF1 drives both proliferation and adipocyte differentiation of hemangioma stem cells (18), while IGF2 elevated in proliferative IH, promotes HemSC growth and adipogenesis via upregulation of PPARγ-CEBP axis (19). Clinically, IH patients exhibit significantly higher serum levels of IGF-2 compared to healthy controls, correlating with disease severity (20). Additionally, the circular RNA *circATP5SL* accelerates IH progression by acting as a sponge for miR-873-5p, thereby enhancing *IGF1R* expression (22). These findings collectively underscore the importance of hypoxia-responsive and growth factor signaling pathways in IH pathogenesis.

In this study, we utilized m6A immunoprecipitation microarray (Epitranscriptomic Microarray) combined with RT-qPCR to systematically characterize differential m6A methylation profiles and associated gene expression patterns between proliferative and involuting phases IH tissues. We aimed to elucidate the functional role of m6A modification in hemangiomas pathogenesis and delineated its regulatory effects on critical biological processes including angiogenesis, cellular proliferation, and programmed cell death. These findings may establish a molecular foundation for developing precise diagnostic and therapeutic strategies for IH.

## 2 Materials and methods

### 2.1 Sample selection

This study included patients diagnosed with infantile hemangioma (IH) at Shanghai Ninth People’s Hospital Affiliated to Shanghai Jiao Tong University School of Medicine. A total of six samples were collected: three from the proliferative phase and three from the involuting phase of IHs. The staging of all patients was based on clinical diagnostic criteria and disease progression characteristics, ensuring that the selected samples accurately represented the proliferative and involuting phases of IH. Sample collection strictly adhered to standardized protocols to ensure the consistency and reliability of the experimental data. Tissue samples were immediately snap-frozen in liquid nitrogen after surgical resection or biopsy, and stored at -80 °C to prevent RNA degradation.

### 2.2 RNA extraction

Total RNA was extracted using TRIzol reagent (Sigma-Aldrich, T9424) according to the manufacturer’s instructions. Cells were lysed in 1 mL of TRIzol, and phase separation was performed by adding 200 µL of chloroform, followed by centrifugation at 12,800 rpm for 10 minutes at 4 °C. The aqueous phase was collected, and RNA was precipitated with an equal volume of pre-chilled isopropyl alcohol. After centrifugation, the RNA pellet was washed twice with 75% ethanol, air-dried, and dissolved in RNase-free water. RNA concentration and purity were measured using a NanoDrop 2000 spectrophotometer (Thermo Fisher Scientific), and samples were stored at −80 °C until use.

### 2.3 Reverse transcription and quantitative real-time PCR

The cDNA synthesis was performed using total RNA extracted from tissue samples with the following reagents: RNase Inhibitor (Epicentre), SuperScript™ III Reverse Transcriptase (Invitrogen), 5× RT Buffer (Invitrogen), 2.5 mM dNTP Mix (HyTest Ltd), and primers (Genewiz Biotechnology Co., Ltd). The procedure was conducted using a clean bench (Boxun Medical Equipment Factory), DK-8D Thermostatic Water Bath (Senxin Laboratory Instruments), and GeneAmp PCR System 9700 (Applied Biosystems). First, an annealing mixture containing 1.2 μg RNA, 0.8 μl Oligo(dT)18 primer (0.5 μg/μl), 0.5 μl Random N9 primer (0.5 μg/μl), 1.6 μl dNTP Mix (2.5 mM), and nuclease-free H2O to a final volume of 13.5 μl was prepared and incubated at 65 °C for 5 min followed by immediate placement on ice for 2 min. After brief centrifugation, the reverse transcription reaction was performed by adding 4 μl 5× First-Strand Buffer, 1 μl 0.1 M DTT, 0.5 μl RNase Inhibitor, and 1 μl SuperScript™ III Reverse Transcriptase to the annealed RNA, incubating at 37 °C for 1 min, gently mixing by pipetting, then incubating at 50 °C for 60 min. The reaction was terminated by heat inactivation at 70 °C for 15 min, and the synthesized cDNA was either immediately placed on ice for subsequent use or stored at -20 °C for long-term preservation, with all procedures carried out under RNase-free conditions to prevent RNA degradation.

The synthesized cDNA was subjected to quantitative real-time PCR (qPCR) analysis using the 2X PCR master mix (Arraystar) on a ViiA 7 Real-Time PCR System (Applied Biosystems), with primer sequences designed using Primer 5.0 software. For standard curve generation, a cDNA template expressing the target genes was amplified in a 10 μl reaction mixture containing 5 μl 2X Master Mix, 0.5 μl each of 10 μM forward and reverse primers, and 2 μl cDNA template, using the following cycling conditions: initial denaturation at 95 °C for 10 min, followed by 40 cycles of 95 °C for 10 sec and 60 °C for 60 sec (with fluorescence acquisition). The PCR products were electrophoresed on a 2% agarose gel with ethidium bromide staining to confirm specific amplification, then serially diluted (10-fold gradients from 10–1 to 10-9) to establish standard curves. For sample analysis, each cDNA was tested in duplicate using an 8 μl reaction mixture (5 μl 2X Master Mix, 0.5 μl each primer, and 2 μl nuclease-free water) combined with 2 μl cDNA in 384-well plates. After sealing and brief centrifugation, amplification was performed under identical cycling conditions followed by melt curve analysis (95 °C for 10 sec, 60 °C for 60 sec, then gradual heating to 99 °C at 0.05 °C/sec). For relative quantification, the 2−ΔΔCt method was employed using U6 small nuclear RNA as the endogenous reference gene. Primers used were list in **Supplementary Table S1**.

### 2.4 RNA m6A dot blot

Dot blot analysis was performed to detect m^6^A RNA modifications. Total RNA (2 μL per sample) was denatured at 65 °C for 5 minutes to disrupt secondary structures and immediately chilled on ice. RNA samples were then spotted onto Immobilon-Ny^+^ nylon membranes (Merck Millipore, Cat# INYC00010) and UV-crosslinked using a UV crosslinker (Ningbo Xinzhi, Model 03-II). After crosslinking, membranes were gently agitated for 5 minutes and washed to remove unbound RNA. The membranes were then blocked in 10 mL of blocking buffer (5% non-fat milk powder; Beyotime, Cat# P0216) in 1× PBS (Biosharp, Cat# BL320A) containing 0.1% Tween-20 (Beyotime, Cat# ST1726) for 1 hour at room temperature with gentle shaking. Subsequently, membranes were incubated overnight at 4 °C with 5 mL of primary antibody dilution buffer containing anti-m^6^A antibody (Abcam, Cat# ab284130, 1:250 dilution, 2 μg/mL). Following three washes in PBST (PBS with 0.1% Tween-20), membranes were incubated for 1 hour at room temperature with HRP-conjugated goat anti-rabbit IgG secondary antibody (Abclonal, Cat# AS014, 1:10,000 dilution, 20 ng/mL). After three additional washes (10 minutes each), chemiluminescent detection was performed using 3 mL of Immobilon Western Chemiluminescent HRP Substrate (Millipore, Cat# WBKLS0500) at room temperature in the dark for 5 minutes. Dot signals were visualized and recorded using a fully automated chemiluminescent imaging system (Tanon, Model 5200). RNase-free water (Beyotime, Cat# R0021) and NanoDrop 2000 (Thermo Fisher Scientific) were used throughout to ensure RNA purity and quantification.

### 2.5 Methylated RNA immunoprecipitation-qPCR

m^6^A RNA immunoprecipitation (MeRIP) was performed to enrich m^6^A-modified RNA transcripts. A total of 1–3 μg of RNA mixed with m^6^A spike-in control was denatured at 65 °C for 5 minutes and immediately cooled on ice. The immunoprecipitation reaction (300 μL) contained 27 μL RNA, 60 μL 5× IP buffer (50 mM Tris-HCl, pH 7.4; 750 mM NaCl; 0.5% NP-40), 3 μL RNase inhibitor, 2 μL anti-m^6^A antibody (e.g., Abcam), and 210 μL RNase-free water, and was incubated at 4 °C for 2 hours with gentle rotation. Separately, 20 μL of mouse IgG-conjugated magnetic beads were washed twice with 1× IP buffer, blocked with 0.5% BSA in IP buffer at 4 °C for 2 hours, and washed again. Blocked beads were added to the RNA–antibody mixture and incubated overnight at 4 °C. The next day, beads were collected using a magnetic rack and washed three times with 500 μL IP buffer (containing 1:1000 RNase inhibitor), followed by two washes with wash buffer (100 mM Tris-HCl, pH 7.4; 50 mM NaCl; 0.1% NP-40), each for 10 minutes. Elution was performed with 200 μL of elution buffer (100 mM Tris-HCl, pH 7.4; 1 mM EDTA; 0.05% SDS) containing 4 μL Proteinase K and 2 μL RNase inhibitor at 50 °C for 1 hour. RNA from both input and IP samples was extracted using phenol–chloroform, precipitated with 3 M sodium acetate and ethanol, and dissolved in 20 μL RNase-free water for downstream applications. The enriched RNA obtained from MeRIP was reverse-transcribed into cDNA and subjected to quantitative real-time PCR (RT-qPCR) to assess the relative abundance of m^6^A-modified transcripts. Primers used were listed in **Supplementary Table S2**. Gene-specific primers were used to amplify target regions, and expression levels were normalized to corresponding input RNA using the following formula:


$$\%input=\frac{2^{-Ct\;MeRIP}}{2^{-Ct\;MeRIP}+2^{-Ct\;Supernatant}}\times 100\%$$

### 2.6 RNA m6A methylation epitranscriptomic microarray assay

The quality of total RNA was assessed using a NanoDrop ND-1000 spectrophotometer for concentration and purity, and RNA integrity was evaluated with an Agilent 2100 Bioanalyzer or by MOPS gel electrophoresis. All results were documented in a Sample QC report. For RNA m^6^A immunoprecipitation (MeRIP), total RNA was incubated with anti-N^6^-methyladenosine (m^6^A) antibody. The immunoprecipitated fraction (“IP”) contained m^6^A-enriched RNAs, while the supernatant (“Sup”) represented unmodified RNAs. Both IP and Sup RNA samples were amplified into complementary RNAs (cRNAs) and labeled using the Arraystar Super RNA Labeling Kit. The IP-derived cRNAs were labeled with Cy5 dye, and the Sup-derived cRNAs with Cy3 dye. Equal amounts of Cy5- and Cy3-labeled cRNAs were mixed and hybridized to the Arraystar Human mRNA & lncRNA Epitranscriptomic Microarray (8×60K, Arraystar) at 65 °C for 17 hours using an Agilent Hybridization Oven. Following hybridization and washing, slides were scanned using the Agilent G2505C Microarray Scanner to obtain fluorescence signal intensities for further analysis.

### 2.7 Epitranscriptomic microarray data analysis

Raw data were extracted using Agilent Feature Extraction software. Probes with “P” (present) or “M” (marginal) QC flags in at least three samples were retained for further analysis. Cy5-labeled IP signal intensities were normalized using internal RNA spike-in controls. The normalized signal, representing the relative abundance of m^6^A modification, was defined as the “m^6^A quantity” for each transcript and was calculated as:


m6A quantity=IP(Cy5 normalized intensity).

Differentially m^6^A-methylated mRNAs, lncRNAs, and other non-coding RNAs were identified by comparing m^6^A quantity across samples using fold-change and statistical significance thresholds. Hierarchical clustering and heatmap visualization were performed to examine methylation patterns among samples.


$$IP_{Cy5\;normalized\;intensity}=\text{log}2\left(IP_{Cy5\;raw}\right)-\text{Average}\left[\text{log}2\left(IP_{spike-in\_Cy5\;raw}\right)\right]$$

### 2.8 Gene expression level analysis

Meanwhile, the expression level for a transcript was calculated based on the IP (Cy5-labelled) and Sup (Cy3-labelled) normalized intensities using the following formula:


$$Gene\;Expression\;Level=IP_{Cy5\;normalized\;intensity}+\;Sup_{Cy3\;normalized\;intensity}$$

$$IP_{Cy5\;normalized\;intensity}=\text{log}2\left(IP_{Cy5\;raw}\right)-\text{Average}\left[\text{log}2\left(IP_{spike-in\_Cy5\;raw}\right)\right]$$

$$Sup_{Cy3\;normalized\;intensity}=\text{log}2\left(Sup_{Cy3\;raw}\right)-\text{Average}\left[\text{log}2\left(Sup_{spike-in\_Cy3\;raw}\right)\right]$$

### 2.9 GO enrichment analysis and pathway analysis

To further explore the biological functions of the differentially expressed genes (DEGs) and differentially methylated genes (DMGs) and their potential role in hemangioma progression, Gene Ontology (GO) enrichment analysis and Kyoto Encyclopedia of Genes and Genomes (KEGG) pathway analysis were performed. GO Enrichment Analysis: The clusterProfiler R package was used for GO enrichment analysis, which annotates the DEGs and DMGs across three categories: biological process (BP), molecular function (MF), and cellular component (CC). This study primarily focused on the BP category to explore key biological processes related to angiogenesis, cell proliferation, inflammation regulation, and immune responses in both the proliferative and involuting phases of hemangiomas. KEGG Pathway Analysis: The KEGG database was used to perform pathway enrichment analysis, identifying key signaling pathways involved in hemangiomas at different stages. Enrichment analysis was conducted using Fisher’s exact test, with Benjamini-Hochberg (BH) correction applied, and a significance threshold of P < 0.05.

### 2.10 Statistical analysis

All statistical analyses were performed using R software (version 4.4.2). RNA-Seq data were analyzed for differential expression using DESeq2, with selection criteria of an adjusted P-value < 0.05 and log2 FC > 1 or < -1. GO enrichment analysis and KEGG pathway analysis were performed using the ClusterProfiler R package, with BH correction to control the false discovery rate. For the statistical analysis of m6A methylation levels, MeRIP-Seq data combined with high-throughput sequencing were analyzed using Fisher’s exact test or DESeq2 to assess the significance of m6A modification differences, with an adjusted P-value < 0.05 as the threshold for statistical significance. Data visualization for all experiments was performed using GraphPad Prism and R ggplot2, to ensure clarity and interpretability of the results.

## 3 Results

### 3.1 Regulation of HIF1A-IGF signaling and m6A RNA methylation in IH stages

**Figure 1:**
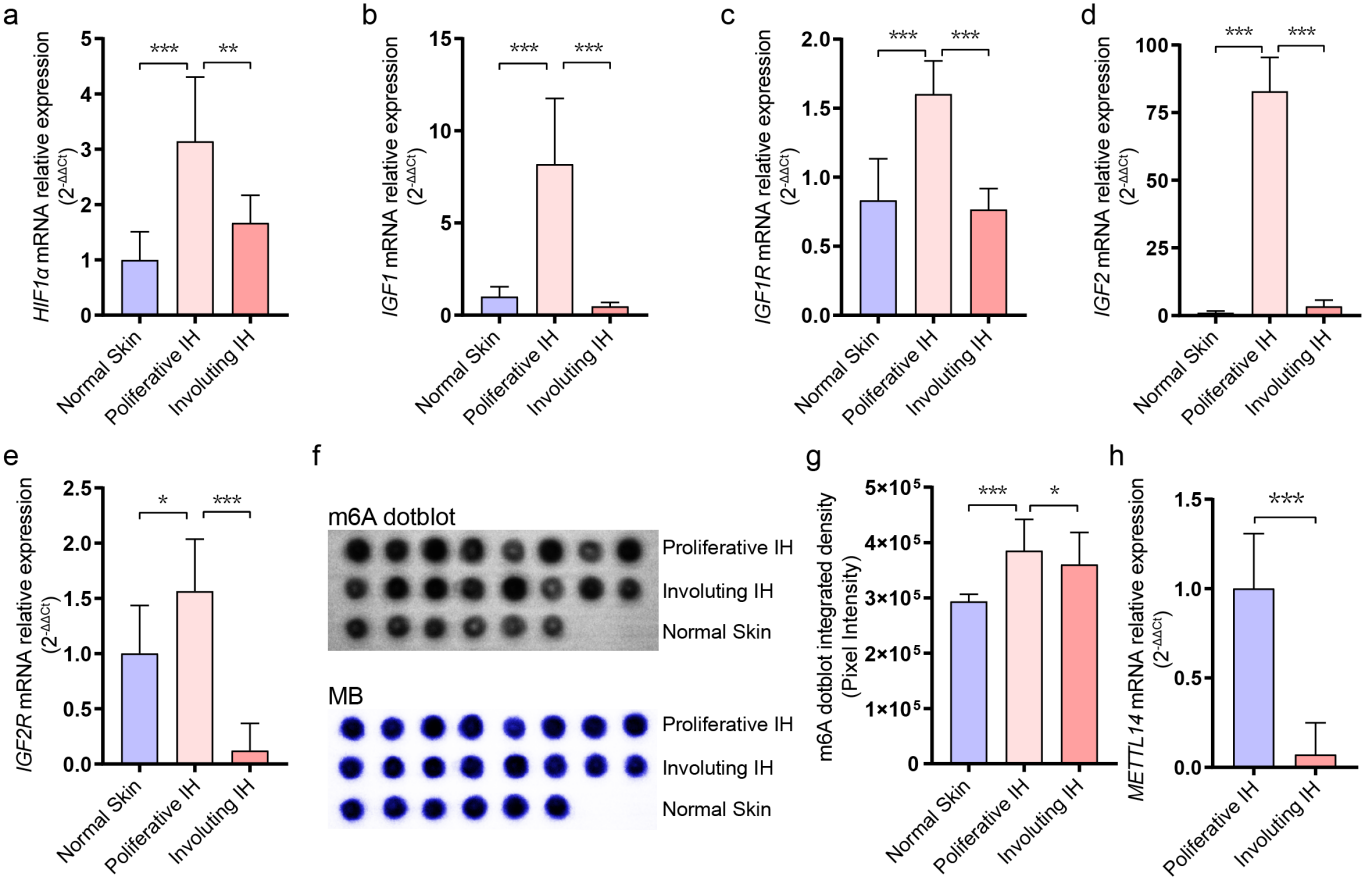
Dynamic changes in RNA m^6^A levels during infantile hemangioma progression. **(A)** RT-qPCR analysis of HIF1A expression levels. **(B)** RT-qPCR analysis of IGF1 expression levels. **(C)** RT-qPCR analysis of IGF1R expression levels. **(D)** RT-qPCR analysis of IGF2 expression levels. **(E)** RT-qPCR analysis of IGF2R expression levels. **(F)** m^6^A modification levels were assessed using dot-blot analysis. **(G)** Quantification of dot blot grayscale intensity using ImageJ software. **(H)** RT-qPCR analysis of METTL14 expression levels. * means p-value < 0.05, ** means p-value < 0.01, *** means p-value < 0.001.

Previous studies have confirmed elevated expression of *HIF1A*, *IGF1*, and *IGF2* in hemangioma tissues. However, their status in involuting IHs has not been explored. Using RT-qPCR, we quantitatively analyzed these genes in normal skin, proliferative IHs, and involuting IHs. The results showed that *HIF1A*, *IGF1*, *IGF1R*, *IGF2*, and *IGF2R* were significantly upregulated during the proliferative phase compared to normal tissue (**Figures 1A–E**), while their expression levels declined during the involuting phase, compared to the proliferative stage (**Figures 1A–E**). These findings further supported the association between these gene expressions and hemangioma progression, corroborating previous reports.

RNA m6A modification has been reported to positively regulate cellular proliferation, yet its role in hemangioma involution remains unexplored. We performed dot blot assays to assess global m6A levels in RNA extracted from normal skin, proliferative hemangiomas, and involuting hemangiomas (**Figure 1F**). We observed that m6A modification was most abundant in the proliferative phase, with a declining trend during involution phase (**Figure 1G**). RT-qPCR further revealed a marked downregulation of METTL14, an m6A writer protein, during the involuting tissues (**Figure 1H**), while the expression levels of other RNA m6A relative genes had no significant differentiation (**Supplementary Figure S1**), suggesting that m6A modification may be involved in regulating hemangioma progression. METTL14’s selective downregulation (**Figure 1H**) suggested METTL14 may preferentially modify pro-proliferative transcripts in IH, unlike METTL3’s broader substrate range.

### 3.2 Transcriptomic profiling reveals active remodeling during IH involution

**Figure 2:**
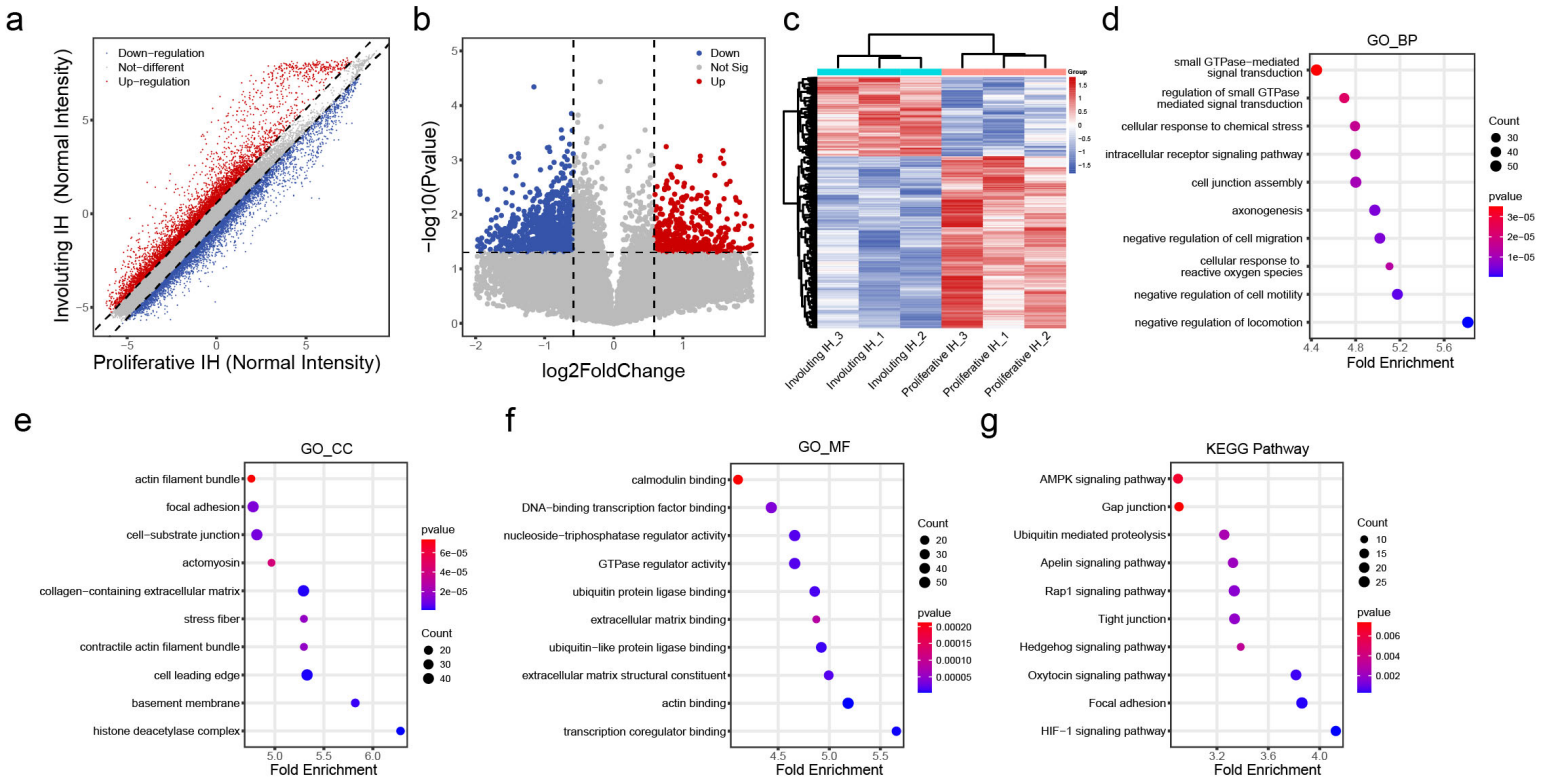
Differential transcriptomic profiles in the involuting phase of hemangiomas. **(A)** Scatter plot of differentially expressed transcripts: red dots indicate upregulated genes and blue dots indicate downregulated genes in the involuting phase; the dashed line indicates fold change = 1.5. **(B)** Volcano plot of differentially expressed transcripts: red dots indicate significantly upregulated genes and blue dots indicate significantly downregulated genes in the involuting phase; the horizontal line represents p value = 0.05, and the vertical lines represent fold change = 1.5. **(C)** Heatmap clustering of differentially expressed transcripts. **(D)** GO Biological Process (BP) enrichment analysis of differentially expressed transcripts: x-axis shows z-score; color indicates p value (bluer = smaller p value); dot size reflects the number of genes enriched in each term. **(E)** GO Cellular Component (CC) enrichment analysis of differentially expressed transcripts (as above). **(F)** GO Molecular Function (MF) enrichment analysis of differentially expressed transcripts (as above). **(G)** KEGG pathway enrichment analysis of differentially expressed transcripts (as above).

To identify regulatory factors involved in hemangioma involution, gene expression profiling was conducted on involuting hemangioma samples (n = 3). After filtering low-expressing genes, the expression profile analysis revealed massive transcriptomic remodeling during hemangioma involution, with 5,371 upregulated and 5,084 downregulated genes (fold-change >1.5, **Figure 2A**, **Supplementary Table S3**). After statistical refinement (pvalue < 0.05), 442 significantly upregulated and 956 downregulated genes (**Figure 2B**) were identified, demonstrating a strong bias toward gene suppression during regression. This suggested that involuting IH is an active, coordinated process, potentially involving post-transcriptional regulation. The clear separation of proliferative from involuting samples in clustering analysis (**Figure 2C**) reinforced that these changes were biologically meaningful and stage-specific.

Biological Process (BP) enrichment analysis demonstrated coordinated changes in GTPase-mediated signal transduction, intracellular receptor signaling, and cell junction assembly, suggesting a shift from proliferative to stabilization programs (**Figure 2D**). Cellular Component (CC) analysis revealed striking enrichment for actin filament bundles, focal adhesions, and basement membrane components, indicating profound cytoskeletal reorganization and extracellular matrix (ECM) remodeling (**Figure 2E**). Molecular Function (MF) analysis highlighted calmodulin binding, GTPase regulator activity, and ECM structural constituents, consistent with altered mechanotransduction and cell-ECM interactions (**Figure 2F**). KEGG pathway analysis reinforced these findings, showing involvement of HIF-1 signaling, AMPK pathway, and gap junction regulation (**Figure 2G**). These findings provide a roadmap for future mechanistic studies, particularly regarding the transcriptional drivers orchestrating this transition and potential therapeutic targets to accelerate involution.

### 3.3 Comprehensive analysis of m6A epitranscriptomic remodeling during IH involution

**Figure 3:**
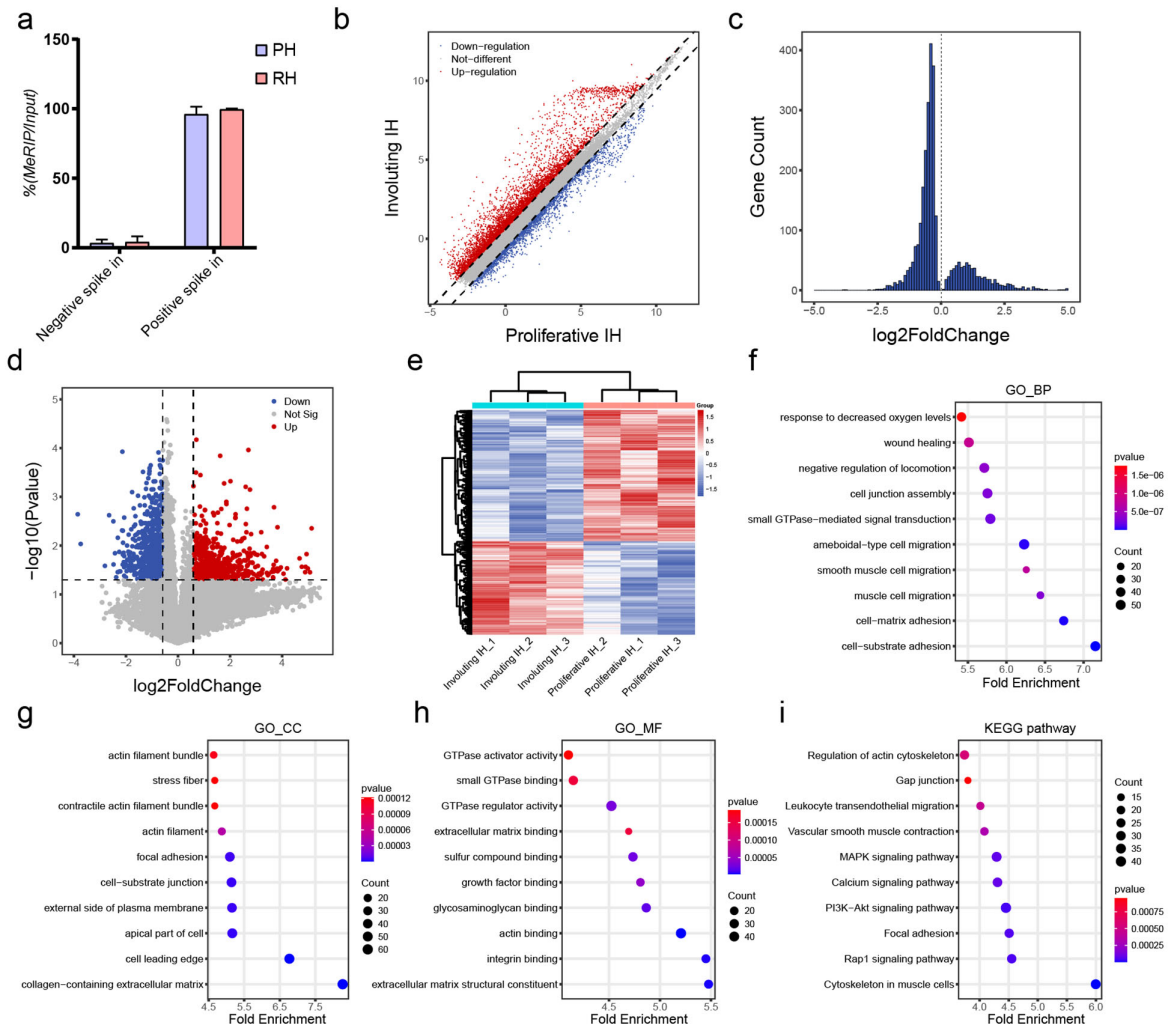
Differential m^6^A methylation profiles of mRNAs in involuting hemangiomas. **(A)** Percentage of MeRIP/Input of Negative and Positive control. **(B)** Scatter plot of differentially methylated mRNAs: red dots indicate increased m^6^A methylation and blue dots indicate decreased methylation in the involuting phase; dashed line = fold change 1.5. **(C)** Histogram of differentially methylated mRNAs with p value < 0.05. **(D)** Volcano plot of differentially methylated mRNAs: red = significantly increased m^6^A methylation, blue = significantly decreased; horizontal line = p value = 0.05; vertical lines = fold change = 1.5. **(E)** Heatmap clustering of differentially methylated mRNAs. **(F)** GO BP enrichment analysis for differentially methylated mRNAs: x-axis = z-score; color = p value (bluer = smaller p); dot size = number of genes. **(G)** GO CC enrichment analysis for differentially methylated mRNAs. **(H)** GO MF enrichment analysis for differentially methylated mRNAs. **(I)** KEGG pathway enrichment analysis for differentially methylated mRNAs.

To elucidate transcript-specific changes in m6A methylation during hemangioma progression, we conducted m6A-RIP chip assays on tissues from proliferative and involuting hemangiomas (n = 3). As shown in **Figure 3A**, the m^6^A enrichment levels of both positive and negative spike-in controls exhibited similar trends between the proliferative IH and involuting IH groups, indicating the robustness and consistency of the experimental procedure. We identified a total of 54,832 m6A-modified transcripts, including 41,263 mRNAs, 10,492 lncRNAs and 1,431 pri-miRNAs, 943 pre-miRNAs, 684 snoRNAs, 19 snRNAs (**Supplementary Table S4**), collectively referred to as small non-coding related RNAs (sncRNAs).

Among protein-coding transcripts, 5,915 mRNAs exhibited increased m6A levels, while 2,396 showed decreased modification (fold-change >1.5; **Figure 3B**). Using a p-value <0.05 as the cutoff, we identified 2,133 upregulated and 704 downregulated m6A-modified mRNAs (**Figure 3C**), indicating a trend toward decreased modification. With both criteria (fold-change >1.5 and p < 0.05), we identified 820 significantly downregulated and 583 significantly upregulated mRNAs (**Figure 3D**). Clustering of these transcripts based on m6A levels distinctly separated proliferative and involuting IH samples, indicating the epitranscriptomic signatures reflect disease states (**Figure 3E**).

Functional annotation of differentially methylated mRNAs uncovered their enrichment in response to hypoxia, cell junction assembly, and cell–matrix adhesion (GO-BP; **Figure 3F**), components such as actin filament bundles and collagen-containing ECM (GO-CC; **Figure 3G**), and functions including GTPase activator activity and integrin binding (GO-MF; **Figure 3H**). DMGs were enriched in KEGG pathways included actin cytoskeleton regulation, gap junctions, and MAPK signaling (**Figure 3I**). These suggest m6A modifications are intricately involved in cellular migration and adhesion mechanisms. Notably, the asymmetric distribution of m6A changes (more hypomethylated transcripts) aligns with METTL14 downregulation (**Figure 1H**), suggesting writer-specific control over involution-related mRNAs and cooperates with HIF1A/IGF suppression (**Figures 1A-E**) to promote vascular quiescence.

### 3.4 RNA m6A modifications influence gene expression

**Figure 4:**
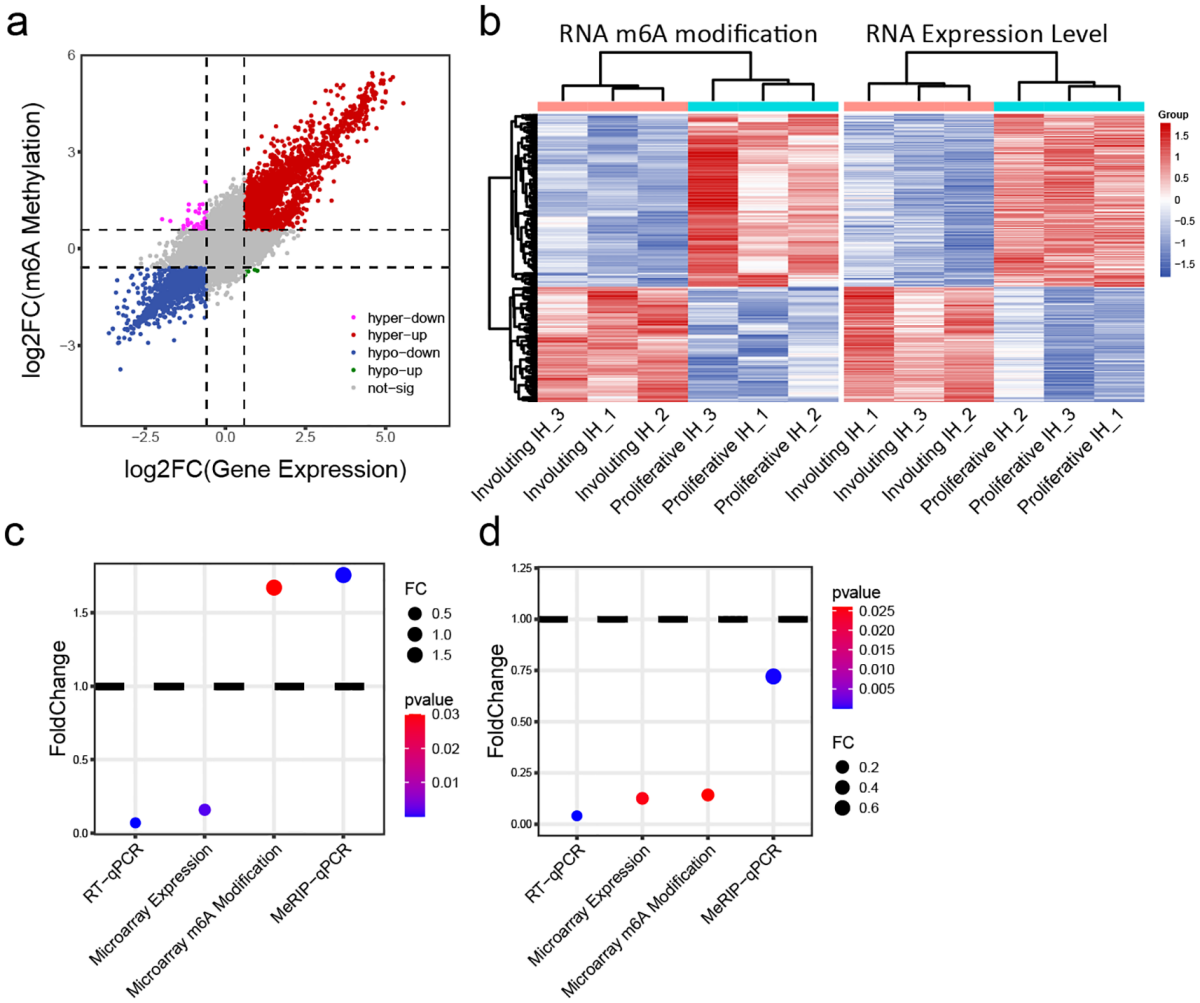
Integrative analysis of m^6^A modification and mRNA expression levels. **(A)** Joint analysis of m^6^A methylation fold change and gene expression fold change: x-axis = expression fold change; y-axis = m^6^A modification fold change. **(B)** Heatmap of transcripts showing both significantly different m^6^A modification and expression levels: left panel = m^6^A heatmap; right panel = expression heatmap. **(C)** Changes in IGF1 expression and m^6^A methylation in involuting hemangiomas compared to proliferative hemangiomas: y-axis = fold change; color = p value. **(D)** Changes in IGF2 expression and m^6^A methylation in involuting hemangiomas compared to proliferative hemangiomas: y-axis = fold change; color = p value.

Our comprehensive analysis of m6A-mediated gene regulation in hemangioma progression reveals a sophisticated epitranscriptomic mechanism that operates in both transcript-specific and phase-dependent manners. Extensive studies suggest that m6A modifications regulate transcript stability including m6A may promote degradation (23) or enhance stability (24). While global correlation analysis demonstrated an overall positive association between m6A levels and transcript abundance (**Figure 4A**), suggesting a predominant stabilizing role of m6A modifications during vascular remodeling, our focused investigation of IGF signaling components uncovered a more complex regulatory network. Genes with both significantly altered expression and m6A modification (fold-change >1.5, p < 0.05) were clustered (**Figure 4B**), again distinguishing proliferative IH and involuting IH groups. The distinct behaviors of IGF1 (showing increased m6A modification but decreased expression during involution) and IGF2 (exhibiting coordinated reduction in both m6A levels and expression) (**Figures 4C, D**) highlight critical aspects of m6A biology in hemangioma progression. These findings suggested that the epitranscriptomic regulation of hemangioma progression involves a delicate balance between global trends and gene-specific exceptions, with important implications for developing stage-specific therapeutic interventions that target both transcriptional and post-transcriptional control nodes in vascular remodeling.

### 3.5 m6A methylation of non-coding RNAs

**Figure 5:**
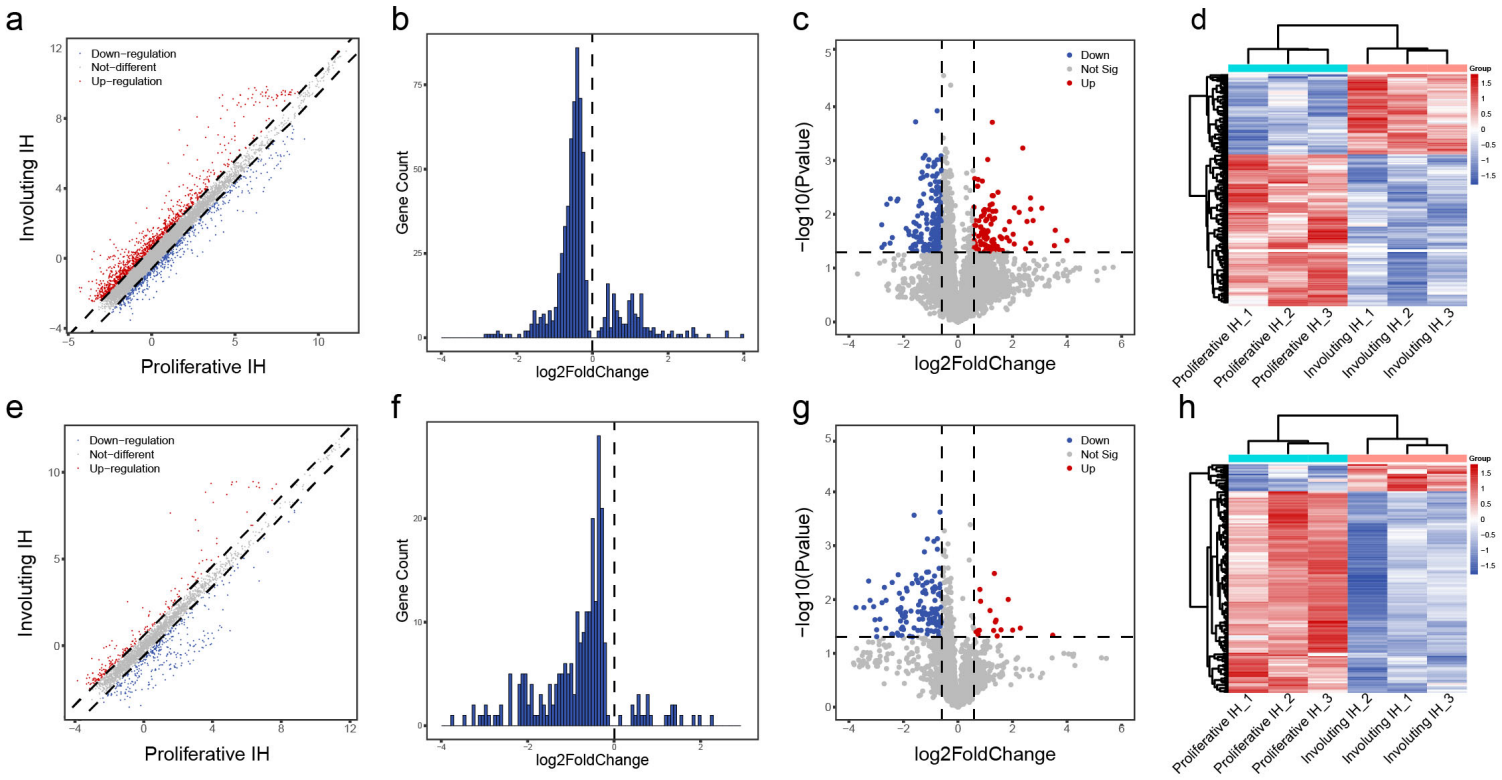
Differential m^6^A methylation profiles of lncRNAs and other small RNAs. **(A)** Scatter plot of differentially methylated lncRNAs: red = increased m^6^A in involuting phase, blue = decreased; dashed line = fold change = 1.5. **(B)** Histogram of lncRNAs with p value < 0.05. **(C)** Volcano plot of differentially methylated lncRNAs: red = significantly upregulated m^6^A, blue = downregulated; p value = 0.05; fold change = 1.5. **(D)** Heatmap clustering of differentially methylated lncRNAs. **(E)** Scatter plot of differentially methylated small ncRNAs. **(F)** Histogram of differentially methylated small ncRNAs with p value < 0.05. **(G)** Volcano plot of differentially methylated small ncRNAs. **(H)** Heatmap clustering of differentially methylated small ncRNAs.

Previous studies have reported that non-coding RNAs play a critical role in the progression of IH. Kun et al. discovered that lncRNA NEAT1 promotes tumorigenesis in IH by regulating FOSL1 expression through the ceRNA mechanism (25). Zhou and colleagues identified lncRNA TUG1 as a key regulator of IH development via the miR-137/IGFBP5 axis (26). Thus, we examined the differential m6A methylation in non-coding RNAs. Among 10,492 lncRNAs and 3,077 small ncRNAs (1,431 pri-miRNAs, 943 pre-miRNAs, 684 snoRNAs, and 19 snRNAs) analyzed, we observed a predominant loss of m6A modifications during the proliferative-to-involuting transition, with 198 lncRNAs showing significant hypomethylation versus only 107 hypermethylated species (fold-change >1.5, p<0.05; **Figures 5A-C**). This global reduction was even more pronounced in small ncRNAs, where 126 species exhibited decreased methylation compared to just 17 with increased marks (**Figures 5E-G**), suggesting particularly important roles for m6A in regulating small RNA function during vascular regression. The distinct clustering patterns between proliferative and involuting phases (**Figures 5D, H**) demonstrate that ncRNA m6A signatures serve as molecular fingerprints of disease state.

The striking bias toward m6A loss, particularly among small regulatory RNAs, suggests involution may involve suppressive methylation processes that could be harnessed therapeutically, potentially through targeted modulation of METTL14 to accelerate vascular normalization.

In summary, global RNA m6A methylation significantly decreases during hemangioma involution. Differentially expressed and m6A-modified genes participate in cell–cell adhesion, cell–ECM interactions, and proliferation-related signaling pathways, offering new insights into the mechanisms underlying hemangioma regression. These findings may pave the way for identifying novel therapeutic targets by modulating m6A modifications.

## 4 Discussion

Infantile hemangioma (IH) stands as the most common benign vascular tumor in infants and demonstrates a unique biphasic life cycle featuring rapid proliferation followed by spontaneous involution (1). While clinicians have well documented this progression pattern, the molecular mechanisms underlying these changes, particularly those involving epigenetic regulation, remain incompletely characterized (2). Florica et al. reveals a 0.11% prevalence of infantile hemangiomas (IH), strongly associated with prematurity, *in vitro* fertilization, maternal conditions (hypertension, anemia, hypothyroidism), and placental complications (placenta previa, twin pregnancy) (27). K Zhang and his colleagues found sex-based disparities in IH presentation: males favor localized/superficial lesions, whereas females show higher segmental involvement, ulcer risk, and post-propranolol rebound (28). Our study offers novel insights by demonstrating that m6A RNA methylation serves as a critical regulatory mechanism governing IH progression through distinct epitranscriptomic programs operating during proliferative versus involuting phases.

During the proliferative phase, we observed coordinated upregulation of both gene expression and m6A methylation, particularly in genes associated with cell cycle progression and angiogenesis. This finding aligns with emerging evidence showing m6A modifications can enhance mRNA stability and translation efficiency of proliferative transcripts in other biological systems (29). The specific identification of cell cycle regulators as major m6A targets suggests a mechanism through which epitranscriptomic modifications maintain the proliferative capacity of IH endothelial cells. This phenomenon may explain the clinical observation of rapid tumor growth during early infancy, as m6A-mediated stabilization of key growth factors could create a positive feedback loop driving vascular expansion.

The transition to involution featured global reduction in m6A levels, particularly on apoptosis-related transcripts. This finding contrasts with cancer models where m6A loss typically promotes malignancy (30), suggesting IH represents a unique model of physiological rather than pathological vascular regression. The specific downregulation of METTL14 we observed may drive this process by reducing m6A deposition on survival factors, thereby permitting programmed vascular remodeling. This hypothesis finds support in recent work demonstrating METTL14’s role in maintaining vascular integrity (31). The specific downregulation of METTL14 (rather than other writers like METTL3 or WTAP) suggests a potentially selective mechanism for m^6^A reduction during involution. This parallels findings in liver regeneration (32), where METTL14 specifically regulated hepatocyte differentiation.

Several important implications emerge from our findings. The biphasic m6A dynamics suggest temporal regulation of “writer” and “eraser” enzymes that could become therapeutic targets. While our study provides compelling evidence for m6A’s role in IH progression, certain limitations require acknowledgment. The sample size, though comparable to other rare disease studies, may affect statistical power for detecting subtle changes. Additionally, our gene microarray approaches cannot resolve cell-type specific effects in these heterogeneous tumors. Future studies employing single-cell m6A sequencing (m6A-scRNA-seq) could address this limitation while providing spatial context to the observed modifications.

## 5 Conclusion

Our study establishes m6A methylation as a central regulator of IH progression and provides a comprehensive resource for understanding epitranscriptomic regulation in vascular biology. These findings not only advance our fundamental knowledge of IH pathogenesis but also identify multiple testable hypotheses for therapeutic development. The unique biology of IH, positioned between physiological and pathological vascular remodeling, makes it an ideal model for studying fundamental principles of vascular growth control.

## Data Availability

The original contributions presented in the study are included in the article/**Supplementary Material**, further inquiries can be directed to the corresponding author/s.
