# Supplementary figures and images for: Dynamic alterations in m6A RNA methylation profiles during involution of infantile hemangiomas
Source: Front Oncol. 2025 Sep 4;15:1652621. doi: 10.3389/fonc.2025.1652621 (PMC12443581; doi:10.3389/fonc.2025.1652621)

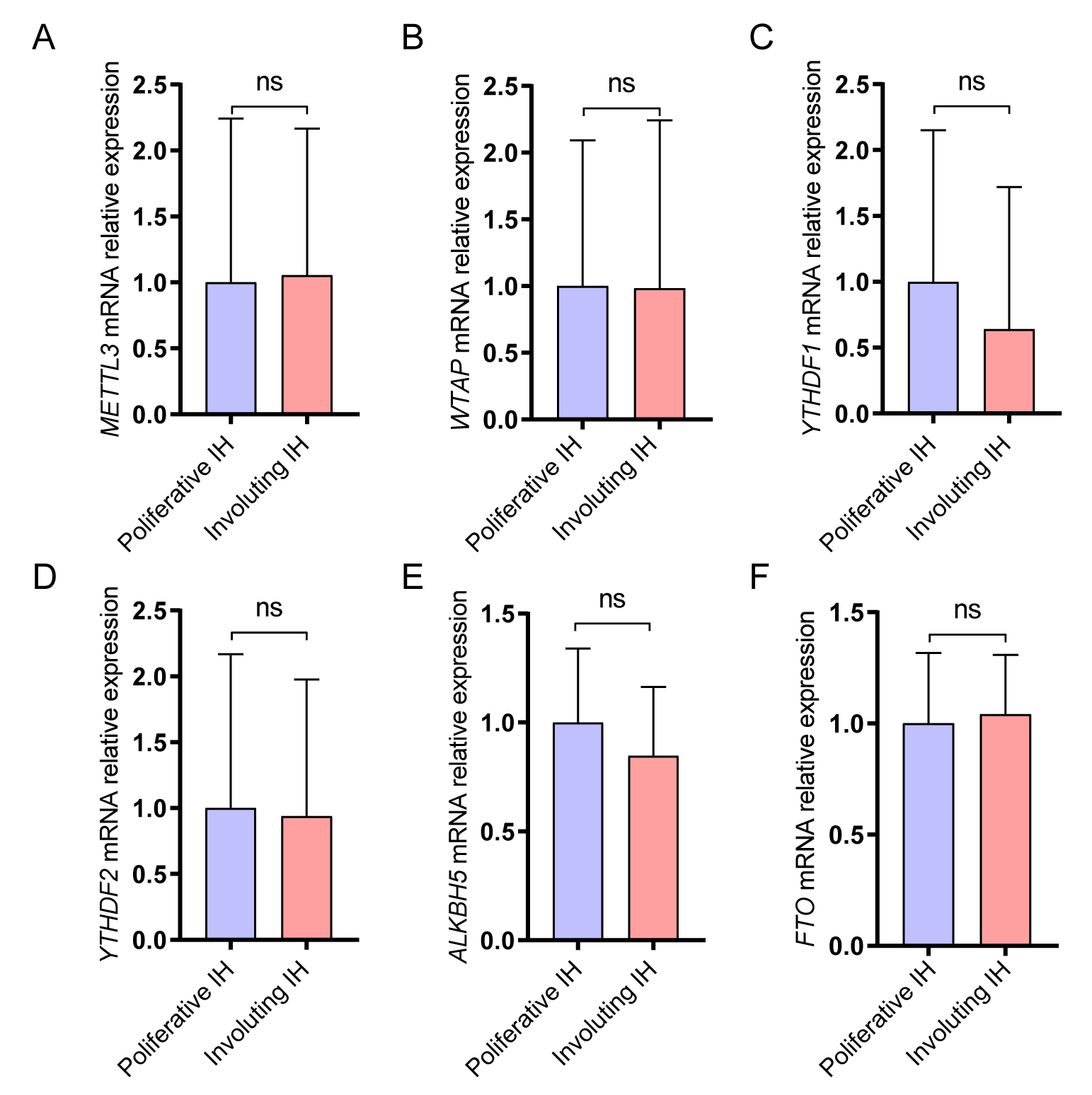

Supplement: Supplementary file 1 [file Image1.tif]
